# Long-Term Outcomes and Prognostic Factors of Medial Open Wedge High Tibial Osteotomy for Medial Compartment Knee Osteoarthritis or Osteonecrosis

**DOI:** 10.3390/jcm14072294

**Published:** 2025-03-27

**Authors:** Yuji Arai, Shuji Nakagawa, Atsuo Inoue, Yuta Fujii, Ryota Cha, Kei Nakamura, Kenji Takahashi

**Affiliations:** 1 Department of Sports and Para-Sports Medicine, Graduate School of Medical Science, Kyoto Prefectural University of Medicine, Kyoto 602-8566, Japan; y123arai@koto.kpu-m.ac.jp; 2 Department of Orthopaedics, Graduate School of Medical Science, Kyoto Prefectural University of Medicine, Kyoto 602-8566, Japan; a-inoue@koto.kpu-m.ac.jp (A.I.); y-fujii@koto.kpu-m.ac.jp (Y.F.); r-cha@koto.kpu-m.ac.jp (R.C.); keinaka2@koto.kpu-m.ac.jp (K.N.); t-keji@mbox.kyoto-inet.or.jp (K.T.)

**Keywords:** MOWHTO, osteoarthritis, BMI, JLCA, alignment, Fujisawa point

## Abstract

**Background/Objectives:** Medial open wedge high tibial osteotomy (MOWHTO) has led to favorable clinical results since the introduction of locking plates. Surgical indications, techniques, and postoperative alignment are crucial for achieving favorable clinical outcomes. This study analyzed the clinical outcomes of patients after >5 years of post-MOWHTO follow-up to identify the influential factors. **Methods:** Thirty-nine patients (48 knees) underwent MOWHTO for medial compartment knee osteoarthritis or -necrosis and were followed up for >5 years. The targeted postoperative % mechanical axis (%MA) was 62.5% (Fujisawa point). The Japanese Orthopaedic Association (JOA) Knee Disease Outcome Criteria score; Kellgren–Lawrence classification; hip-knee-ankle, medial proximal tibial, mechanical lateral distal femoral, and joint line convergence angles (JLCA); and %MA were evaluated preoperatively, at implant removal, and at the final follow-up. Total knee arthroplasty (TKA) was the survival endpoint. Uni- and multivariate analyses were performed to identify the factors influencing survival rates. **Results:** The mean JOA score improved from preoperative to implant removal and was sustained at 102 months. Four of the 48 knees required TKA, resulting in a 10-year survival rate of 82%. Body mass index, preoperative JLCA, and Δ%MA influenced the post-MOWHTO survival rate. The Δ%MA was significantly greater in the group with a %MA < 62.5% at implant removal. **Conclusions:** MOWHTO with a target %MA of 62.5% yielded favorable long-term outcomes. Additionally, preoperative obesity and high joint instability negatively influenced post-MOWHTO survival. Furthermore, a postoperative %MA of < 62.5% is associated with difficulty maintaining stable alignment and an increased risk of conversion to TKA.

## 1. Introduction

Articular cartilage consists of water and an extracellular matrix composed of aggrecan, type II collagen, hyaluronic acid, and a small number of chondrocytes. Regenerating articular cartilage is challenging due to its absence of nerves, blood vessels, and lymph vessels [1]. From a histological perspective, articular cartilage comprises distinct superficial, middle, deep, and calcified layers. The deep layer is also referred to as the calcified layer; beneath it lies the directly continuous subchondral bone. Articular cartilage is a hyaline cartilage that possesses exceptional compression, shearing, and tension properties. It can withstand mechanical loads, minimize friction, and reduce the force transmitted to the subchondral bone [2]. However, when exposed to excessive mechanical stress, articular cartilage exhibits metabolic abnormalities including decreased production of extracellular matrix components such as aggrecan and type II collagen by chondrocytes at the molecular level, increased chondrocyte apoptosis, and extracellular matrix destruction by a number of proteolytic enzymes. These changes ultimately lead to its degradation.

Osteoarthritis (OA), the most prevalent joint disease, is a degenerative disorder primarily characterized by articular cartilage degeneration. Current estimates indicate that more than 250 million individuals worldwide are affected by OA [3]. As the global population ages, the prevalence of OA is also expected to increase. Knee OA, in particular, has been identified as a major contributor to disability in both middle-aged and elderly populations, significantly impacting activities of daily living, often manifesting as knee pain and gait disturbances. This condition imposes a substantial financial and economic burden on healthcare systems and the broader economy [4]. The primary risk factors for knee OA include obesity and lower-limb malalignment such as varus or valgus deformities. Excessive loading on the articular cartilage is responsible for cartilage wear and degeneration [5]. Severe impairment of the hoop function of the meniscus due to a medial meniscus posterior root tear reportedly results in excessive loading on the articular cartilage, increasing the risk of rapid progression of knee osteoarthritis and osteonecrosis [6]. Consequently, the treatment of these diseases requires reducing the excessive loading on the articular cartilage and meniscus.

Radiography, magnetic resonance imaging (MRI), and ultrasonography are commonly used to diagnose OA. In recent years, vibration arthrography has been proposed as a new, inexpensive, and non-invasive screening method for cartilage damage [7,8,9] that enables the early detection and treatment of OA. Conservative treatment is typical for early-stage OA. Diet and exercise therapy are the cornerstones of treatment, while drug therapy, encompassing the oral administration of acetaminophen and non-steroidal anti-inflammatory drugs and the intra-articular injection of hyaluronic acid and corticosteroids, has also been employed [10]. In recent years, biotherapies such as platelet-rich plasma and stem cell therapies have also been used, with evidence of their ability to provide adequate pain relief and enhance patient function. However, the pathogenesis of OA is intricate and not fully understood, and issues such as the absence of an established drug delivery system for chondrocytes preclude these treatments from suppressing articular cartilage degeneration. In cases in which symptoms persist or cartilage degeneration progresses, surgical treatment is preferred. The most common surgical interventions include osteotomy and artificial joint replacement. High tibial osteotomy (HTO) is considered a conventional surgical procedure that reduces the medial stress by shifting the load axis from the medial to the lateral side, thereby reducing pain. Two surgical approaches have been employed to treat medial compartment overload in varus knees: medial open wedge high tibial osteotomy (MOWHTO) and lateral closing wedge high tibial osteotomy (LCWHTO). The traditional approach is LCWHTO, which involves partial resection of the fibula and wedge-shaped resection of the lateral side of the proximal tibia. However, with the advent of locking plates and mechanically robust artificial bones, MOWHTO has gained popularity because it allows the opening of the medial side of the proximal tibia [11]. The advent of MOWHTO surgical techniques and implants has facilitated early weight-bearing as a relatively simple surgical technique uncomplicated by peroneal nerve palsy, a potential complication of LCWHTO. Preservation of the pes anserinus at the osteotomy site further facilitates its minimally invasive nature [12]. MOWHTO has also demonstrated favorable short- and midterm outcomes [13,14]. Moreover, its use enables the possible long-term avoidance of total knee arthroplasty (TKA). Conversely, in MOWHTO, patient background factors that influence postoperative outcomes include age, sex, weight, OA progression, deformity etiology, and joint instability. Other factors associated with MOWHTO success include target alignment, surgical technique, and increased patellofemoral joint load owing to its low postoperative position [15,16]. Other potential concerns include excessive increases in the inclination of the articular surface and posterior tibial slope. Although many unknown factors of MOWHTO persist, its indications for surgery, surgical technique, and postoperative alignment are crucial factors in achieving favorable clinical outcomes. The identification of factors that exert a detrimental influence on the clinical outcomes of MOWHTO will facilitate the achievement of enhanced long-term results through the selection of appropriate patients and surgical techniques. Here, we sought to analyze the clinical outcomes of patients who could be followed up for >5 years after MOWHTO and identify the influential factors.

## 2. Materials and Methods

### 2.1. Participants

This retrospective study analyzed consecutive cases of medial knee OA or idiopathic knee osteonecrosis for which MOWHTO was performed at our hospital or one of three affiliated institutions between September 2005 and September 2015. The indications for MOWHTO included having exhibited resistance to conservative treatment for a period exceeding 3 months, primarily in cases of medial compartment lesions, and a demonstrated satisfactory range of motion. No restrictions were imposed on sex, body weight, age, OA grade, degree of lower-limb deformity, or anterior cruciate ligament degeneration. Cases with significant degeneration in the lateral compartment or patellofemoral joint, symptoms originating from these areas, flexion contractures ≥ 10°, or low activity levels were excluded. Thus, the MOWHTO was performed for 42 patients (52 knees). Of them, 39 patients (48 knees) for whom follow-up was possible for >5 years were included in this study. The sample included 9 male (11 knees) and 30 female (37 knees). The imaging protocol included coronal, lateral, and axial radiographs of both knees and full-length standing radiographs complemented by plain MRI scans. The results of the imaging tests were evaluated by the respective surgeons. Seven males and 23 females underwent unilateral knee surgery, while 2 males and 7 females underwent bilateral knee surgery. The mean patient height was 160 cm (range, 144–183 cm), mean weight was 67 kg (range, 43–103 kg), mean body mass index (BMI) was 26 kg/m^2^ (range, 18–35 kg/m^2^), mean age was 55 years (range, 29–70 years), and mean postoperative follow-up period was 102 months (range, 63–181 months). The mean follow-up period from MOWHTO to implant removal was 20 months (range, 11–49 months). Surgical procedures were performed by 4 surgeons, with a minimum of 10 years of experience. Surgeon Y.A. operated on 33 patients (41 knees), surgeon S.N. operated on 3 patients (4 knees), surgeon R.T. operated on 2 patients (2 knees), and surgeon H.T. operated on 1 patient (1 knee). A total of 42 knees (34 patients) were operated on at our hospital, while 6 knees (5 patients) were operated on at 3 other hospitals. The medial compartment of the knee was affected by osteoarthritis in 41 knees (KL 1, 2 knees; KL 2, 15 knees; KL 3, 20 knees; KL 4, 4 knees), and spontaneous osteonecrosis was the underlying cause in 7 knees (stage 2, 2 knees; stage 3, 1 knee; stage 4, 4 knees) (Table 1).

### 2.2. Surgical Procedure

Each surgical procedure was performed with the patient in a supine position under general anesthesia. Arthroscopy was initially performed, followed by intraarticular procedures. Medial partial resection was performed in 23 patients (27 knees), lateral discoid lateral meniscus saucerization in 1 patient (1 knee), autologous osteochondral bone grafting in 5 patients (5 knees), and microfracture in 1 patient (1 knee). Following arthroscopy, an incision was made on the proximal medial tibia, and an oblique osteotomy was performed from the medial side approximately 4 cm distal to the articular surface toward the fibular head. The osteotomy was opened to achieve a postoperative % mechanical axis (%MA) of 62.5% (Fujisawa point) in accordance with the preoperative plan. In 9 patients (10 knees), autologous iliac bone was grafted into the open osteotomy, while in 30 patients (38 knees), β tricalcium phosphate was used to fill the area. A Tomofix Plate^®^ (Depuy Synthes, Oberdorf, Switzerland) was used in 38 patients (47 knees), while a Puddu Plate^®^ (Arthrex Inc., Naples, FL, USA) was employed in a single knee. In 3 patients (3 knees), the contralateral implant was removed concurrently with the osteotomy. The mean operative time was 2 h and 28 min (range: 1 h and 27 min to 5 h and 16 min). The estimated mean blood loss was <345 mL. Sixteen patients (20 knees) had a history of smoking. According to the Takeuchi classification [17], no fractures were observed in 29 patients (32 knees). Type I fractures were identified in 13 patients (16 knees); no type II or III fractures were documented.

### 2.3. Evaluation

The Japanese Orthopaedic Association (JOA) Knee Disease Outcome Criteria score, knee extension and flexion angles, osteoarthritis staging (Kellgren–Lawrence [KL] classification), hip–knee–ankle (HKA) angle, medial proximal tibial angle (MPTA), mechanical lateral distal femoral angle (mLDFA), joint line convergence angle (JLCA), and %MA were analyzed preoperatively, at implant removal, and at the final follow-up. The HKA angle was defined as the angle between the mechanical axis of the femur and tibia in the coronal plane. Specifically, varus alignment was designated as negative, whereas valgus alignment was designated as positive. The difference between %MA at the final follow-up and at implant removal was considered Δ%MA to evaluate loss of correction. Preoperatively, cartilage morphology was scored in the medial femoral, medial tibial, lateral femoral, and lateral tibial condyles mainly using T2-weighted SPIR images and PD-weighted SPIR images on an eight-point scale [18]. Articular cartilage was evaluated in 38 patients (47 knees) who underwent arthroscopy at the initial surgery and at implant removal, and was analyzed using the International Cartilage Repair Society (ICRS) classification. Knees that required conversion to total knee arthroplasty (TKA) because of pain or osteoarthritis progression were included in the TKA conversion group, whereas the others were included in the survival group.

### 2.4. Statistical Analysis

Data are expressed as mean ± SD and were analyzed using EZR3.3.3 (Saitama Medical Center, Jichi Medical University), a graphical user interface for R (The R Foundation for Statistical Computing, version 2.13.0). In all analyses, values of *p* < 0.05 were defined as statistically significant. The statistical analyses were performed to assess the correlations among sex, age, BMI, KL classification, HKA, MPTA, JLCA, %MA, and Δ%MA. Uni- and multivariate analyses were also performed. The intraclass correlation coefficients were calculated using a two-way random model. The interobserver reliabilities for the HKA, MPTA, JLCA, and %MA measurements were 0.986, 0.908, 0.789, and 0.989, respectively. The intraobserver reliabilities of observer 1 were 0.996, 0.922, 0.87, and 0.995 for the HKA, MPTA, JLCA, and %MA measurements, respectively, while those of observer 2 were 0.99, 0.949, 0.844, and 0.992, respectively. Pre- and postoperative ICRS classifications were analyzed using the chi-squared test.

## 3. Results

### 3.1. Evaluation of Clinical Outcome and X-Ray Images

This study observed changes in various parameters preoperatively, at implant removal, and at the final follow-up. The mean postoperative JOA score was 63.0, with knee extension and flexion averages of −2.8° and 136.1°, respectively. Other parameters (HKA angle, MPTA, mLDFA, JLCA, and %MA) were also recorded. At the time of implant removal, an improvement in the JOA score (92.3) was observed. Furthermore, substantial alterations in the HKA angle, MPTA, and %MA were observed compared to the preoperative values. At the final follow-up, the mean JOA score was 88.6, indicating no significant difference from the scores at implant removal. Significant improvements were observed in several parameters, including JOA scores, HKA angle, MPTA, and %MA, which remained stable until the final follow-up. In contrast, mLDFA and JLCA changed negligibly over time (Table 2).

### 3.2. Evaluation of Articular Cartilage Using MRI

The morphology of the articular cartilage of the medial femoral, medial tibial, lateral femoral, and lateral tibial condyles was evaluated on preoperative MRI using the eight-point Whole-Organ Magnetic Resonance Imaging Score system. In more than 70% of cases, the medial femoral and medial tibial condyles exhibited four or more locations of cartilage damage. Conversely, the lateral femoral and lateral tibial condyles exhibited a score of 0 in over 90% of cases, with minimal to no cartilage damage observed (Table 3).

### 3.3. Evaluation of Articular Cartilage Using Arthroscopy

A comparative analysis of the ICRS grading of the articular cartilage of the medial femoral, medial tibial, lateral femoral, and lateral tibial condyles at the preoperative and implant removal points revealed no significant changes in any of the examined areas (Table 4).

### 3.4. Evaluation of Survival Rate and Influencing Factors

**Figure 1:**
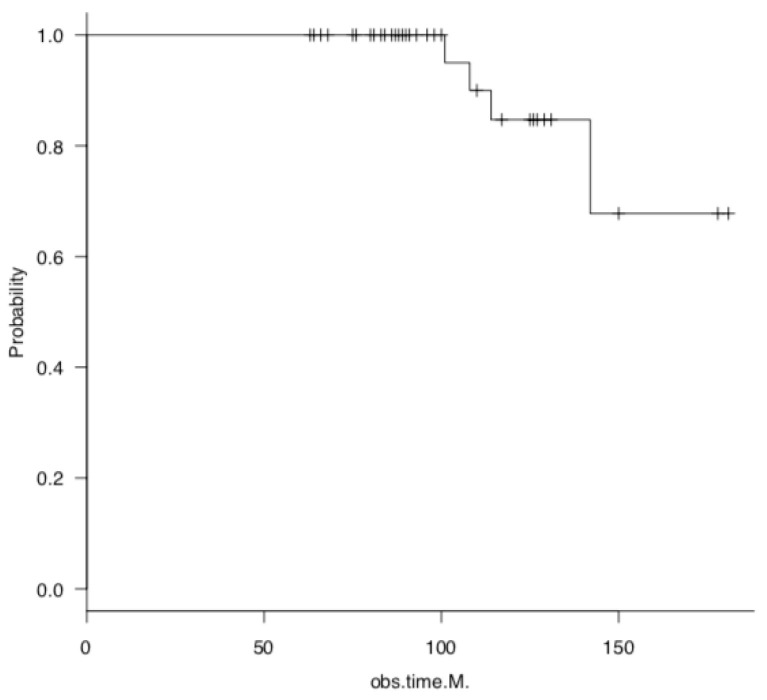
Survival analysis after open wedge high tibial osteotomy, with total knee arthroplasty as endpoint, using the Kaplan–Meier method. The + mark on the line of the survival curve plot indicates the point at which the data was censored.

**Figure 2:**
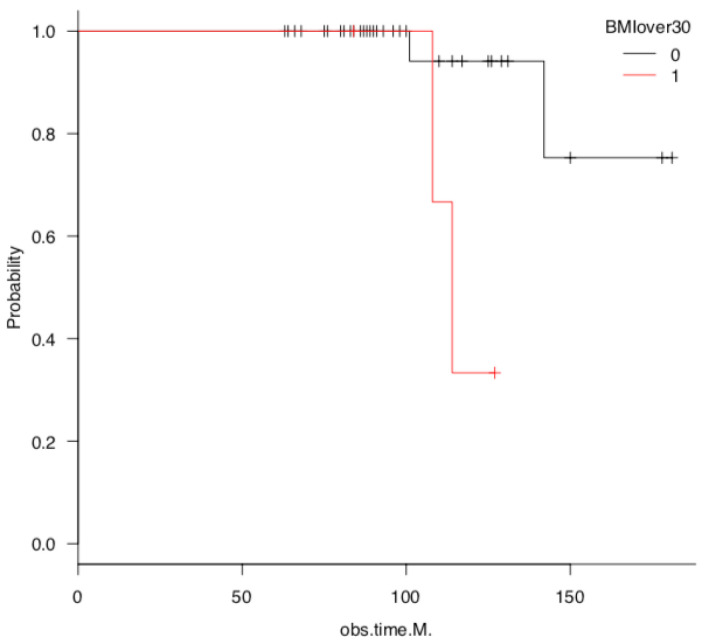
Survival analysis after open wedge high tibial osteotomy, with total knee arthroplasty as endpoint, according to body weight. The + mark on the line of the survival curve plot indicates the point at which the data was censored.

Survival rate was calculated as the endpoint at which TKA conversion was performed or deemed necessary. The survival group comprised 44 knees (10 males, 34 females), whereas the TKA conversion group comprised 4 knees (all from females). The 5- and 10-year survival rates were 100% and 82%, respectively (Figure 1). Patients with a BMI ≥ 30 kg/m^2^ exhibited a lower long-term survival rate than those with a BMI < 30 kg/m^2^ (Figure 2).

The present study compared the survival and TKA conversion rates between the two groups. The mean ages of the two groups were similar (55.4 and 55.0 years). A nonsignificant intergroup difference was observed in KL classification, and OA severity was equivalent between the groups. A comprehensive analysis of preoperative, implant removal, and final follow-up parameters, including HKA, MPTA, JLCA, and %MA, revealed significant variations in BMI, final follow-up HKA, JLCA across different stages, and %MA and Δ%MA on a univariate analysis (Table 5). Significant factors, including BMI, preoperative JLCA, and %MA, were identified in a multivariate analysis (Table 6).

### 3.5. Comparison Between %MA < 62.5 Group and ≥62.5 Group

A comparison of the 22 knees with a %MA < 62.5% and the 26 knees with a %MA ≥ 62.5% at the time of implant removal revealed significant differences in Δ%MA, in addition to %MA at the time of implant removal and at the final follow-up (Table 7).

## 4. Discussion

This study demonstrated that a MOWHTO with a target %MA of 62.5% yields favorable long-term outcomes. In addition, preoperative obesity and high joint instability negatively influenced post-MOWHTO survival. Furthermore, a postoperative %MA of <62.5% was associated with difficulty in maintaining stable alignment and an increased risk of conversion to TKA.

Historically, LCWHTO has yielded the most favorable long-term outcomes for medial-compartment knee osteoarthritis, as Coventry’s technique is the inaugural approach utilized for this indication. Constantin et al. conducted a prospective study of 100 patients who underwent LCWHTO. When conversion to TKA was used as the endpoint, the 20-year survival rate was 44%. The factors that had a positive impact on improving survival were age < 55 years, BMI < 30 kg/m^2^, and absence of serious symptoms [19]. Sasaki et al. conducted a survey of 120 knees subjected to LCWHTO and found that the 15-year survival rate was 92.5%, with obesity and high preoperative symptom severity identified as risk factors for requiring TKA [20]. Ishizuka et al. conducted a survey of 56 knees that underwent LCWHTO and found that the 10-, 20-, and 35-year survival rates were 83.8%, 75.9%, and 75.9%, respectively. The analysis revealed that a BMI ≥ 25 and a femorotibial angle < 185° affected TKA conversion [21].

Conversely, given the relatively recent introduction of MOWHTO, the number of studies examining its long-term survival, sample size, and follow-up period is considerably smaller than those examining LCWHTO. In an early report on MOWHTO with a large sample size, a study of 245 knees, with a target postoperative alignment of 183–186°, was conducted in 2001. The 5-, 10-, and 15-year survival rates were 94%, 85%, and 68%, respectively, with an endpoint of TKA conversion [22]. A meta-analysis conducted by Kim et al. reviewed 23 studies of MOWHTO and LCWHTO with a minimum 5-year follow-up period. The survival rates after conversion to TKA were 95.1% and 91.6% at 5 and 10 years, respectively. Notably, the survival rate at 10 years was higher in the MOWHTO versus LCWHTO group [23]. Keenan et al. reported a 10-year survival rate of 65% and a 15-year survival rate of 55% in 111 patients who underwent MOWHTO [24]. In a comparative study of the long-term outcomes of MOWHTO and UKA, Jin et al. [25] observed that the survival rates at 10 years were 96.2% and 91.6%, respectively. In a large study of 556 patients conducted by Primeau et al., the revision rate from MOWHTO to TKA was 5% at 5 years and 21% at 10 years [26]. In a small study by Lau et al. spanning 13 years, the 10-year survival rate of patients treated with MOWHTO was 87% [27]. According to a systematic review and meta-analysis of 59 studies (5162 patients) by Loke et al., conversion to TKA occurred in 4.5%, 8.3%, and 11.2% of cases at <5, 5–10, and >10 years, respectively [28]. Thus, despite differences in study design, sample size, and surgical technique, the survival rates for MOWHTO are approximately 95% at 5 years, 65–92% at 10 years, and 55–75% at 15 years or longer. In our small study, four of the 48 knees required TKA, with a 5-year survival rate of 100% and a 10-year survival rate of 82%. These findings are consistent with the survival rates observed in the aforementioned clinical trials and generally indicate good long-term results.

As a target postoperative alignment for HTO, the criterion that the weight-bearing line passes through 62–66% of the tibial plateau width, as recommended by Dugdale et al. in 1992, has been widely utilized [29]. This criterion is consistent with the finding that the weight-bearing line passes laterally to the lateral intercondylar ridge of the tibia in alignment with the HKA at an angle of 3–5° and includes the Fujisawa point, which was used as our target alignment point. Conversely, numerous approaches to target alignment have been proposed in recent years. Atkinson et al. utilized MRI T2 mapping to qualitatively evaluate articular cartilage pre- versus postoperatively and reported that correcting the alignment to be closer to neutral versus excessive valgus alignment enhanced cartilage quality without compromising the lateral compartment [30]. Kim et al. reported favorable clinical outcomes in valgus cases with a mechanical axis of 0–3° by performing cartilage repair surgery involving microfracture, autologous cultured chondrocyte transplantation, and stem cell transplantation with concomitant MOWHTO [31]. However, inadequate correction and residual postoperative varus alignment are also associated with a poor prognosis and the progression of medial compartment osteoarthritis of the knee [32]. In this study, regardless of preoperative lower-limb alignment, the mean %MA at the time of implant removal was 62.2%, which approached the target value of 62.5%. Furthermore, significant improvements in clinical scores were maintained at the final follow-up. The 10-year survival rate was 82%, and a univariate analysis demonstrated that the alignment at the time of implant removal was associated with postoperative outcomes. A univariate analysis demonstrated that alignment at the time of implant removal was not a risk factor for conversion to TKA. This indicates that MOWHTO, targeting this alignment, is advantageous for achieving a favorable long-term outcome.

Using a musculoskeletal computer model, Kuriyama et al. analyzed the correction of various frontal planes in MOWHTO during walking and squatting. They found that Fujisawa point alignment (%MA, 62.5%) sufficiently loaded the lateral femorotibial joint after MOWHTO, maintaining normal knee kinematics [33]. In this study, no significant changes were observed in the cartilage evaluation using the ICRS classification with arthroscopy, either medially or laterally, pre- versus postoperatively. This suggests that this surgical technique also protects the medial compartment biomechanically and can avoid excessive lateral-side loading. Conversely, the %MA at the final follow-up was 57.8%, lower than that at the time of implant removal, indicating that alignment was inversely related to the long-term outcome of MOWHTO. Additionally, a univariate analysis demonstrated that both %MA and Δ%MA at the final follow-up were associated with an increased risk of conversion to TKA. Furthermore, a multivariate analysis indicated that Δ%MA was an independent predictor of conversion to TKA. Moreover, a comparison of 26 patients with a %MA ≥ 62.5% at the time of implant removal, which included 22 patients who did not reach this threshold, revealed a significantly greater Δ%MA in those who did not reach the threshold. This suggests that if the postoperative %MA is <62.5% in MOWHTO, the risk of conversion to TKA may increase owing to difficulty maintaining stable alignment.

The survival rate after HTO is influenced by several prognostic factors, including sex, age, obesity, and preoperative knee osteoarthritis severity. Regarding sex, some studies have indicated that females are at increased risk, whereas others did not identify any such association [34,35,36,37]. The present study did not identify an association between sex and survival among patients who underwent MOWHTO. Further research is required to ascertain the impact of sex on this outcome. Evidence indicates that older adults aged ≥ 60 years demonstrate comparable performance on short-term tests to younger individuals [38,39,40]. However, a predominant finding suggests that advanced age (specifically, 50–60 years) tends to have a deleterious impact on survival outcomes [41]; in the majority of cases, age 55 years or older is considered a risk factor. In this study, the mean patient age was 55 years, and no age-based effect was observed. The acceptable BMI range is 25–30 kg/m² [41]. In a recent study, Bouguennec et al. [42] demonstrated that a BMI > 25 kg/m² significantly elevated the risk of conversion to TKA; moreover, a higher BMI (>35 kg/m²) further augmented this risk. In the present study, a BMI > 30 kg/m² was also associated with an increased risk of revision TKA, indicating that obesity has a detrimental impact on the long-term outcomes of MOWHTO. Therefore, the indications for MOWHTO in patients with obesity should be carefully evaluated; if indicated, patients should be encouraged to lose weight before undergoing surgery.

Regarding the adverse impact of knee osteoarthritis on HTO, an Ahlbäck classification grade of III or higher with loss of joint space and mild loss of subchondral bone on plain radiographs is considered a risk factor. Cartilage degeneration severity on arthroscopic findings is also a poor prognostic factor [26,27,42,43]. One study examining cartilage damage severity on arthroscopy and the long-term outcomes of MOWHTO found that patients > 40 years of age with advanced cartilage damage and an Outerbridge classification of 3–4 had a higher rate of conversion to TKA [44]. Gkekas et al. reported favorable long-term outcomes for cases of high-severity OA (KL3 and 4), with a survival rate of 91.5% after a mean of 13.6 years [45]. Knee osteoarthritis severity did not appear to influence the MOWHTO outcomes in this study. However, it is possible that this was not identified as a risk factor because of the limited number of cases included in the study; specifically, only four cases of KL 4 with no joint space were included. Conversely, preoperative varus–valgus joint instability may also have an influence. In particular, postoperative alignment adjustment is challenging when MOWHTO is employed to treat patients with advanced knee osteoarthritis who have lost medial articular cartilage and have a large JLCA, resulting in instability [46,47]. As this study identified JLCA as a factor that negatively affects the survival rate of MOWHTO, alternative techniques such as tibial condylar valgus osteotomy [48,49] and double-level osteotomy [50] should be considered in patients with significant preoperative joint instability.

This study is clinically significant in that it demonstrates the importance of evaluating patient weight and knee joint instability in the application of MOWHTO for medial knee joint disease. Furthermore, good long-term results can be obtained with MOWHTO targeting the Fujisawa point.

## 5. Conclusions

BMI and preoperative JLCA are the most significant predictors of conversion to TKA, indicating that long-term joint preservation after MOWHTO is challenging to achieve in patients with preoperative obesity and pronounced varus–valgus joint instability. Furthermore, if the postoperative %MA is < 62.5%, maintaining stable alignment will be challenging, and the risk of conversion to TKA will increase.

### Limitations

This study had several limitations. First, it was retrospective and included a small sample size. Future studies should include a larger patient population or use a multicenter design. Second, this retrospective study examined cases in which MOWHTO was performed with a target alignment of 62.5%; thus, a future prospective study with randomized target alignment is required. Third, patient-based evaluations were not conducted. Fourth, in this study, the medial meniscus was left untreated or only partially resected, and a partial autologous osteochondral graft was performed on the cartilage. Consequently, the combined effects of meniscal repair, centralization, and cartilage regeneration therapies require further investigation in future studies. Fifth, all of the cases in this series that had a cause of deformity on the femoral side also underwent an OWHTO.

## Figures and Tables

**Table 1:** Demographics of the study population (n = 48).

Variable	Value
Diagnosis (osteoarthritis/osteonecrosis)	41/7
Sex (male/female)	11/37
Age (years)	55.4 ± 9.1
BMI (kg/m^2^)	26.0 ± 3.8
Follow-up (months)	102 ± 21

Abbreviations: BMI, body mass index.

**Table 2:** Clinical and imaging data from evaluations performed preoperatively, at implant removal, and at the final follow-up.

Parameters	Preoperative	Implant Removal	Final Follow-Up
KL (1/2/3/4)	5/15/23/5	5/22/20/1	5/17/18/8
JOA score (points)	63.0 ± 11.7 (range, 40 to 80)	92.3 ± 7.1 (range, 75 to 100)	88.6 ± 16.4 (range, 40 to 100)
ROM ext (deg)	−2.8 ± 3.6 (range, −10 to 0)	−1.8 ± 3.3 (range, −10 to 0)	−3.8 ± 5.0 (range, −20 to 0)
flex (deg)	136.1 ± 8.6 (range, 115 to 150)	140.6 ± 7.7 (range, 115 to 150)	137.5 ± 11.3 (range, 110 to 150)
HKA angle (deg)	−6.4 ± 3.1 (range, −13.3 to 0.2)	3.0 ± 3.2 (range, −5.7 to 9.8)	1.8 ± 4.2 (range, −8.2 to 10.1)
MPTA (deg)	84.0 ± 1.8 (range, 79.6 to 87.3)	93.1 ± 3.0 (range, 86.9 to 99.7)	92.5 ± 3.4 (range, 86.6 to 100.4)
mLDFA (deg)	87.7 ± 2.4 (range, 83.6 to 93.3)	88.2 ± 2.1 (range, 84.1 to 93.0)	88.3 ± 2.2 (range, 84.0 to 93.2)
JLCA (deg)	3.0 ± 1.8 (range, −0.6 to 7.5)	2.2 ± 1.6 (range, −0.6 to 6.5)	2.6 ± 1.8 (range, −1.3 to 7.2)
%MA (%)	18.6 ± 13.1 (range, −8.6 to 43.7)	62.2 ± 13.8 (range, 25.5 to 85.4)	57.8 ± 17.6 (range, 15.4 to 88.8)

Abbreviations: KL, Kellgren–Lawrence; JOA, Japanese Orthopaedic Association; ROM, range of motion; HKA, hip knee ankle; MPTA, medial proximal tibial angle; mLDFA; mechanical lateral distal femoral angle; JLCA, joint line convergence angle; MA, mechanical axis.

**Table 3:** Eight-point WORMS system used to assess knee cartilage morphology.

WORMS 8-Point Scoring for Cartilage Morphology	Area			
	MFC	MTC	LFC	LTC
0	1 (2.1%)	2 (4.3%)	44 (93.6%)	45 (95.7%)
1	0 (0%)	0 (0%)	0 (0%)	1 (2.1%)
2	6 (12.8%)	12 (25.5%)	2 (4.3%)	1 (2.1%)
2.5	0 (0%)	0 (0%)	0 (0%)	0 (0%)
3	0 (0%)	0 (0%)	0 (0%)	0 (0%)
4	18 (38.3%)	15 (31.9%)	1 (2.1%)	0 (0%)
5	10 (21.3%)	8 (17.0%)	0 (0%)	0 (0%)
6	12 (25.5%)	10 (21.3%)	0 (0%)	0 (0%)

Abbreviations: WORMS, Whole-Organ Magnetic Resonance Imaging Score; MFC, medial femoral condyle; MTC, medial tibial condyle; LFC, lateral femoral condyle; LTC, lateral tibial condyle.

**Table 4:** Arthroscopic ICRS grading of knee cartilage before versus after implant use.

Area	ICRS grading (0/1/2/3/4)		*p* Value
	Preoperative	Implant Removal	
MFC	1/5/13/15/13	1/6/18/15/7	0.61
MTC	1/9/13/10/14	1/14/9/14/9	0.46
LFC	34/11/2/0/0	30/16/1/0/0	0.52
LTC	26/19/2/0/0	18/26/3/0/0	0.23

Abbreviations: ICRS, International Cartilage Repair Society; MFC, medial femoral condyle; MTC, medial tibial condyle; LFC, lateral femoral condyle; LTC, lateral tibial condyle.

**Table 5:** Univariate analysis of the survival versus conversion to total knee arthroplasty groups.

Parameters	Survival Group	TKA Conversion Group	*p* Value
	(n = 44)	(n = 4)	
Sex (male/female)	10/34	0/4	0.57
Age (years)	55.4 ± 9.3	55.0 ± 7.0	0.93
BMI (kg/m^2^)	25.6 ± 3.6	31.3 ± 2.3	<0.01 *
KL (1/2/3/4)	5/15/20/4	0/0/3/1	0.35
HKA angle preoperative (deg)	−6.3 ± 3.1	−7.9 ± 3.6	0.33
implant removal (deg)	3.1 ± 3.2	1.3 ± 2.7	0.28
final follow-up (deg)	2.4 ± 3.9	−3.9 ± 4.7	<0.01 *
MPTA preoperative (deg)	84.0 ± 1.8	84.7 ± 2.4	0.46
implant removal (deg)	93.1 ± 3.1	93.5 ± 0.6	0.8
final follow-up (deg)	92.7 ± 3.4	90.1 ± 3.0	0.15
JLCA preoperative (deg)	2.8 ± 1.6	6.0 ± 1.0	<0.01 *
implant removal (deg)	2.0 ± 1.5	4.5 ± 1.4	<0.01 *
final follow-up (deg)	2.3 ± 1.7	5.6 ± 1.2	<0.01 *
%MA preoperative (%)	19.0 ± 13.0	14.2 ± 13.7	0.49
implant removal (%)	62.9 ± 13.5	55.3 ± 10.4	0.28
final follow-up (%)	59.9 ± 16.1	34.4 ± 20.2	<0.01 *
Δ%MA (%)	3.0 ± 7.4	20.9 ± 13.0	<0.01 *

Abbreviations: BMI, body mass index; KL, Kellgren–Lawrence; HKA, hip knee ankle; MPTA, medial proximal tibial angle; JLCA, joint line convergence angle; MA, mechanical axis. Significance * *p* < 0.05.

**Table 6:** Multivariate regression analysis of factors influencing conversion to total knee arthroplasty.

Parameters	Odds Ratio	95% CI	*p* Value
BMI (kg/m^2^)	1.018	1.001–1.036	0.04 *
Δ%MA (%)	1.011	1.003–1.019	<0.01 *
Pre JLCA (deg)	1.042	1.001–1.084	0.04 *

Abbreviations: BMI, body mass index; MA, mechanical axis; JLCA, joint line convergence angle. Significance * *p* < 0.05.

**Table 7:** Univariate analysis of groups with a percentage mechanical axis of <62.5% versus >62.5% at implant removal.

Parameters	%MA < 62.5	%MA ≥ 62.5	*p* Value
	(n = 22)	(n = 26)	
Sex (male/female)	5/17	5/21	1
Age (years)	54.5 ± 10.1	56.2 ± 8.3	0.52
BMI (kg/m^2^)	26.1 ± 3.8	26.0 ± 3.9	0.97
Follow-up (months)	119.7 ± 55.5	111.0 ± 39.0	0.61
KL (1/2/3/4)	0/8/10/2	3/7/13/3	0.49
%MA preoprattive (%)	16.1 ± 13.2	20.8 ± 12.7	0.22
implant removal (%)	50.2 ± 8.2	72.4 ± 6.5	<0.01 *
final follow-up (%)	42.8 ± 12.7	70.4 ± 9.7	<0.01 *
Δ%MA (%)	7.3 ± 10.7	2.0 ± 7.2	0.047 *

Abbreviations: BMI, body mass index; KL, Kellgren–Lawrence; MA, mechanical axis. Significance * *p* < 0.05.

## Data Availability

The original contributions presented in this study are included in the article. Further inquiries can be directed to the corresponding author.

## Abbreviations

The following abbreviations are used in this manuscript:

%MA	% mechanical axis
BMI	body mass index
FTA	femorotibial angle
HKA	hip–knee–ankle
JLCA	joint line convergence angle
JOA	Japanese Orthopaedic Association
KL	Kellgren–Lawrence
LCWHTO	lateral closing wedge high tibial osteotomy
LFC	lateral femoral condyle
LTC	lateral tibial condyle
MA	mechanical axis
MFC	medial femoral condyle
MTC	medial tibial condyle
mLDFA	mechanical lateral distal femoral angle
MOWHTO	medial open wedge high tibial osteotomy
MPTA	medial proximal tibial angle
MRI	magnetic resonance imaging
OA	osteoarthritis
ROM	range of motion
TKA	total knee arthroplasty
WORMS	Whole-Organ Magnetic Resonance Imaging Score
